# Novel Compliant Scaffold with Specific Design for Venous System: Results of a Porcine Model Study

**DOI:** 10.1155/2018/7312315

**Published:** 2018-01-31

**Authors:** Pierfrancesco Veroux, Alessia Giaquinta, Carla Virgilio, Davide Danilo Zani, Giuliano Ravasio, Vincenzo Ardita, Paola Secchiero, Eugenio Scanziani, Paolo Zamboni, Massimiliano Veroux

**Affiliations:** ^1^Vascular Surgery and Organ Transplant Unit, Department of Surgery, University Hospital of Catania, Catania, Italy; ^2^Veterinary University Hospital, School of Veterinary Medicine, University of Milan, Milan, Italy; ^3^Azienda Polo Veterinario di Lodi, Department of Veterinary Sciences and Public Health, University of Milan, Milan, Italy; ^4^Department of Morphology, Surgery and Experimental Medicine and LLTA Center, University of Ferrara, Ferrara, Italy; ^5^Mouse and Animal Pathology Lab (MAPLab), Filarete Foundation, Milan, Italy; ^6^Vascular Disease Center and Institute of Translational Medicine and Surgery, University of Ferrara, Ferrara, Italy

## Abstract

**Background:**

Stenting has become the first-line treatment of obstructive venous disease because of poor results of balloon angioplasty. This preclinical study aimed to investigate the safety and efficacy profile of a novel compliant venous scaffold (CVS) denominated Petalo CVS, specifically designed for venous diseases.

**Materials and Methods:**

Twelve healthy pigs weighing 90 kg were used to test Petalo CVS. The devices were implanted into the internal jugular veins (IJVs) using a femoral vein percutaneous approach. The safety profile including the success rate of device releasing, anchoring, and positioning was evaluated immediately. Fracture, migration, primary patency, and endothelial response were assessed at 1, 2, 3, and 6 months after the study procedure.

**Results:**

A total of 32 devices were successfully released in both IJVs. No procedure- or device-related complications were reported, and all pigs successfully completed the different scheduled follow-up periods. The primary patency rate was 100%, and no fracture or migration of the device into the brachiocephalic trunk was reported. Histological examination revealed only minimal lesions with minimal or absent inflammatory reaction surrounding the incorporated metallic rods.

**Conclusions:**

This porcine model study showed a promising safety and efficacy profile of Petalo CVS, a novel endovenous device based on specific concepts.

## 1. Introduction

Endovenous balloon angioplasty is used to treat occlusive venous disease. This procedure has a satisfactory safety profile but with low patency and clinical improvement rates. It shows minimum interference of the core nature of venous hemodynamics and related collapsibility. Due to the poor results of balloon angioplasty, stenting is used with increasing frequency in patients with venous chronic obstructive disease. Endovenous stenting results in improved midterm patency compared with balloon dilatation [1–4].

An ideal venous stent design should consider the compliance of the venous system to the hydrostatic pressure, continuously modified by the postural changes of the body. The most recent endovenous stents are mainly derived from arterial stent concepts, with a potentially high radial force and resistance to compression; thus, they fall short of these requirements.

In this porcine model study, the safety, efficacy, and endothelial response of Petalo CVS, a novel nitinol device, specifically based on venous properties, were evaluated.

## 2. Materials and Methods

Twelve healthy pigs were used to test Petalo CVS, a novel specific scaffold based on venous properties. The study was approved by the local ethics committee of University of Milan and the Italian Ministry of Health (MoH). The study was conducted following the guidelines of good laboratory practices and received a grant from the National MoH.

The internal jugular vein (IJV) was used in this study model because of its similarity in size and length to the human IJV. Moreover, it can be easily approached by a femoral vein percutaneous access and monitored using Doppler ultrasound. It also offers a valuable stress site for migration and fracture of the device due to the frequent movements and torsion of the neck, and likely compression by neck muscles. Finally, the IJV can be easily retrieved for macroscopic and histological examinations at the end of the study.

### 2.1. Experimental Venous Device

The prototypes were fabricated by Admedes Schuessler GMBH (Pforzheim, Germany) using a cut-laser nitinol technology. Petalo CVS has two Italian patents and a European patent (PL2914214 (T3), 2017-07-31).

**Figure 1:**
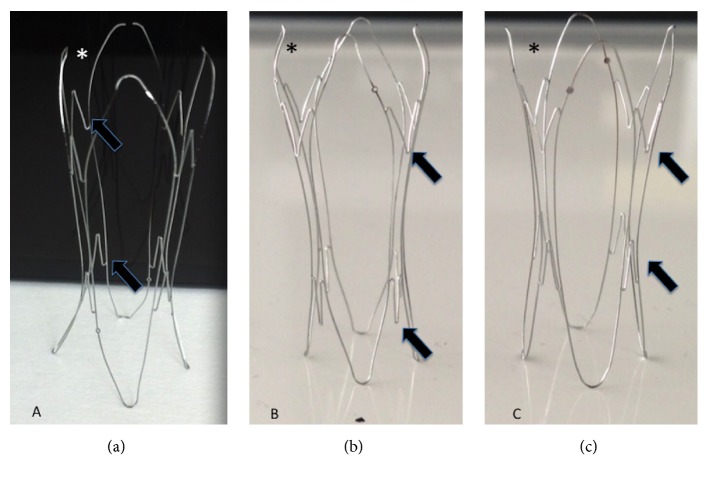
Petalo CVS prototypes: scaffold shape evolutions. In the first prototype (a), the heads of the bridges (arrows) between the modules are directed upwards; in the second prototype (b), the heads are directed downwards; and in the final prototype (c), the heads are in a “kissing” position to minimize the risk of vein perforation and improve the collapsibility of the scaffold. Moreover, in the final prototype, the upper extremities of the modules (*∗*) are oriented with an external configuration to reduce the contact area and minimize the risk of migration.

Petalo CVS has a tubular concave shape with 4 support modules joined by transverse bridges (Figure 1).

The modules are oriented longitudinally and extend along the entire length of the device's body. The concavity of the four modules is oriented toward the vein wall. The support modules are internally empty, without bridges or other internal elements, to minimize the metal structure. Two transversal bridges join the support modules in the central part of the body, leaving the extremity of the modules free and open. The transversal bridges have a gull-wing shape. The joined bridges enhance the conformability of the device and reduce the radial force. The concave shape of the support modules was studied to reduce the contact between the device and vein wall, with the aim of decreasing inflammatory response. Three prototypes with the same shape and length were tested, and the one with higher radial force was chosen. The prototype had proximal (nearest to the heart) and distal diameters of 12 mm and 14 mm, respectively. Two devices with different lengths (38 mm and 48 mm) but the same diameters were used for the study. The longer device had an increased radial force than the shorter one. The study device was designed to provide a scaffold for the venous wall to treat endoluminal defects, stenosis, and extrinsic compression of intracerebral venous sinus and peripheral straight veins. Petalo CVS enables the veins to physiologically adapt its cross-sectional area to the flow and hydrostatic changes by collapsing when pressure and/or flow rate decreases. This avoids the persistent dilatation of the vein wall observed with available venous stents and finally contributes to maintain the physiological compliance of the venous wall.

### 2.2. Animal Model and Study Protocol

Twelve healthy pigs weighing 90 kg were used in the experimental study.

The experimental study was performed in two phases. Phase 1 was defined as the procedure of device delivery, and phase 2 was the procedure of control and harvesting of the target veins. Phase 1 procedures were performed for three consecutive days (four pigs per day). All endovascular procedures were conducted under general anesthesia using a standard protocol and monitoring of cardiac and respiratory parameters. The percutaneous approach to the venous system and catheter venography was similar to that performed normally in humans and already described in a previous paper [5]. In brief, access to the venous system was achieved through a percutaneous antegrade approach of the right common femoral vein under sonographic guidance to avoid accidental arterial puncture. After placement of a 9 F sheath introducer (Boston Scientific, Natick, Massachusetts), an intravenous bolus of 5,000 U of heparin was administered. The right IJV was first cannulated using a 0.035-inch short-angle regular hydrophilic guide wire 260 cm in length (Aquatrack; Cordis, Bridgewater, New Jersey) supported by a BER II diagnostic catheter (4 F, 100 cm; Cordis). The BER II catheter, a straight catheter with a short distal angulation of the tip, was preferred to minimize possible interference with any endoluminal defects of the IJVs. Selective venography of the IJVs was performed by manual injection of a low-viscosity contrast medium (Iomeron 150; Bracco Imaging, Milano, Italy) in anterior-posterior projection. Subsequently, at least one device was inserted in the proximal or medium tract of the IJV or at the site of valve apparatus using a standard pull-back stent delivery system (Video 1). A new phlebography was performed to evaluate the position of the device, the patency of the vein, and time of clearance of contrast medium as parameters of flow characteristics. The same procedure was performed for the left jugular vein. At least two devices with different lengths were inserted for each pig. At the end of the procedure, the inguinal introducer was removed, and light manual compression of the access site was performed. The procedure was now considered complete, and the pig was extubated and placed in the animal house. All pigs received a daily oral dose of 100 mg of acetylsalicylic acid for 1 month. Every minor or major adverse event at the access site, or related to the procedure or devices, was accurately monitored in each pig. Monitoring was performed in all awake pigs and during the postoperative period to avoid adverse events and harm. Phase 2 procedures were performed at different scheduled times: 1 month (4 pigs), 2 months (2 pigs), 3 months (2 pigs), and six months (4 pigs) after phase 1 procedures. All pigs underwent a new endovascular procedure and an open surgery procurement of the target veins using the same anesthetic and endovascular protocols of phase 1 procedures described previously. The correct position of the device (migration) using bone markers, integrity of the scaffold (fracture), and patency of all segments of the IJV including the scaffold were evaluated and documented, together with the clearance of contrast medium (Video 2).

The IJVs were then isolated through a standard cutdown technique to evaluate the elasticity and persistence of compliance of the vein wall using two methods. Manual hydrostatic dilatation of the vein was initially performed by low-pressure injection of sterile solution through a direct puncture of distal IJVs, simulating an increase of the flow volume and pressure. The bed was then moved in Trendelenburg and anti-Trendelenburg positions to evaluate the changes of the cross-sectional area of the veins. Finally, IJVs including the device were removed to examine the macroscopic and microscopic changes of the vein wall in response to the device. Histological examination of the target vein samples was performed at two blinded laboratories.

The surgical specimen included a vein portion of at least 2 cm in length proximally and distally to the device. For the histological analyses, samples were fixed in formalin 10% for 24 h at 4°C and subsequently rinsed in several changes of cold 70% ethanol. The tissues were dehydrated through an alcohol series and then paraffin-embedded using a Shandon Citadel 2000 Tissue Processor. After blocking out, 5 *μ*m thick sections were cut, stained with haematoxylin and eosin (Bio Optica SPA, Milano, Italy), and/or used for immunohistochemistry with the Ab anti-von Willebrand Factor (vWF) (FVIII) (Dako, Carpinteria, CA). In each slide, a negative control was obtained carrying out the immunohistochemistry procedure without the primary antibody. All the sections were acquired with a light microscope (Eclipse TE200 Inverted Microscope; Nikon, Tokyo, Japan). All metallic rods were removed to allow histological processing. For the wall thickness evaluation, different sections were acquired and digitalised with an Aperio ScanScope® slide scanner and the thickness values of the jugular walls were estimated by using the Aperio ImageScope v11.1.2.760 software (Leica Biosystems, Nussloch, Germany). For each tissue section, the total wall thickness was obtained from at least 3 randomly selected areas within the slide. All haematoxylin and eosin-stained jugular vein sections were examined by the study pathologist for semiquantitative and descriptive histopathology and for assessment of inflammatory cells and infiltration of lymphoid tissue around the empty area previously occupied by metallic rods of the device.

The pigs were sacrificed at the end of the procedure under deep general anesthesia with intravenous administration of Tanax. All data related to positioning and follow-up of the endovascular device, including the diagnostic procedures and open surgery harvesting of the target vein, were recorded in an electronic database and hard clinical report. All the study procedures were video recorded. All the videos were stored and classified based on the type and time of the procedure. A brief summary of the most significant moments of each procedure was created to make the final video. Images from all surgical specimens were taken, and all specimens were classified accordingly.

### 2.3. Endpoints

The study evaluated a composite of safety and efficacy endpoints. The safety profile included successful delivery and positioning, rate of migration and fracture of the device, and rupture and bleeding of the target vein. Major adverse events (MAE) were defined as death, severe cardiac arrhythmia, and need for open surgery repair. The primary efficacy endpoints were primary patency of the devices, absence of thrombus of the target IJV, persistent elasticity, and compliance of venous wall. The endothelial response to the device was evaluated during histological examination of the target veins.

## 3. Statistical Analysis

The anatomical characteristics of all pigs and intraoperative and postoperative details were collected in a dedicated spreadsheet (excel software). The collected data were expressed as means ± standard deviation and percentages. For stratified analysis, *χ*^2^ test was used to compare different outcomes, and the associated *P* value < 0.05 was considered significant. Device primary patency, freedom from inner and outer IJV stent graft migration, and survival rates were reported with Kaplan-Meier method using dedicated software (Rstudio package, Version 0.99.902, © 2009–2016 RStudio, Inc., Boston, USA). Curves are displayed up to a value of standard error <.10. A 95% confidence interval was used for all variables of the underlying distribution.

## 4. Results

A total of 12 pigs were included in the study, and the procedures were successfully completed in all pigs. Percutaneous access to the venous system was achieved in 96% of cases (23/24). A cutdown access was requested in one case. The right common femoral vein was used in the majority of cases (23/24). Thirty-two devices were implanted in both IJVs (mean 2.67 ± 0.65): 21 devices, 14–12 × 38 mm; and 11 devices, 14–12 × 48 mm. In 7 IJVs (4 RIJV and 3 LIJV), two devices were implanted: one in the proximal segment of IJV and the other in medium portion. In the remaining 18 cases, a single device was implanted (Table 1).

**Figure 2:**
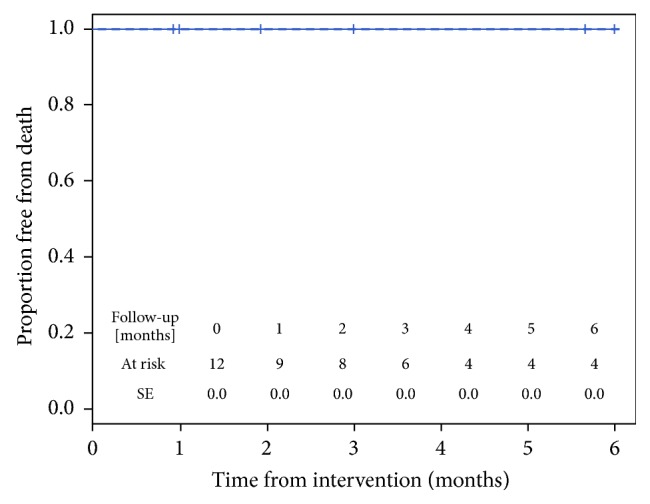
Kaplan-Meier curve demonstrating excellent survival.

All the devices were placed successfully. The positioning was precise, and no immediate migration occurred. In all cases, the devices were able to control the leaflets of the valve. No deaths or MAE were documented, and all pigs reached the scheduled phase 2 procedures (Figure 2).

No significant differences were found between the two devices in terms of patency, reintervention, survival, and inner JV migration rates.

**Figure 3:**
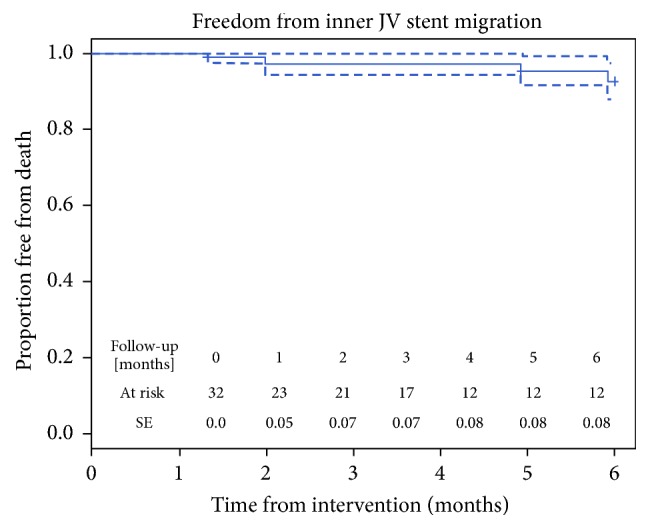
Kaplan-Meier analysis of 6-month freedom from inner IJV stent graft migration demonstrating a low rate of inner migration, confirming the excellent stability of the device.

In phase 2 procedures, no fractures or migration of the scaffold in the brachiocephalic trunk or in the right atrium were reported. In 6 of 32 devices (18.7%), a proximal migration confined to IJV segment was reported. The freedom from inner IJV stent migration rate was 90.6% at 1 month, 83.4% at 3 months, and 78.5% at 6 months from intervention (Figure 3).

**Figure 4:**
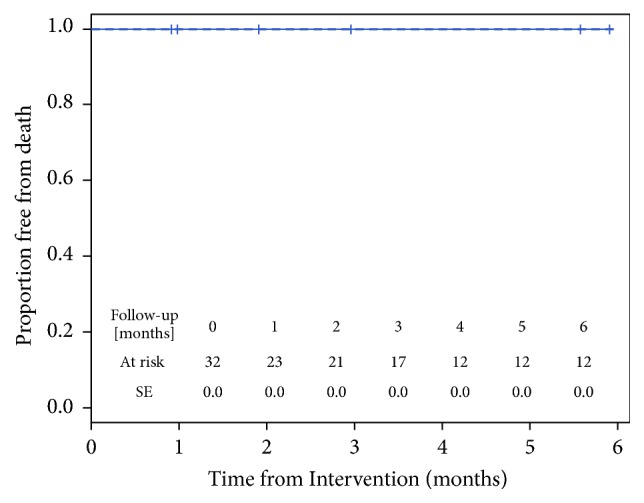
Kaplan-Meier analysis of primary patency. All devices were patent at the scheduled follow-up times.

Despite the discontinuation of antiplatelet therapy after the first month, all veins and devices were patent (primary patency of 100%) at the scheduled phase 2 procedures (Figure 4).

**Figure 5:**
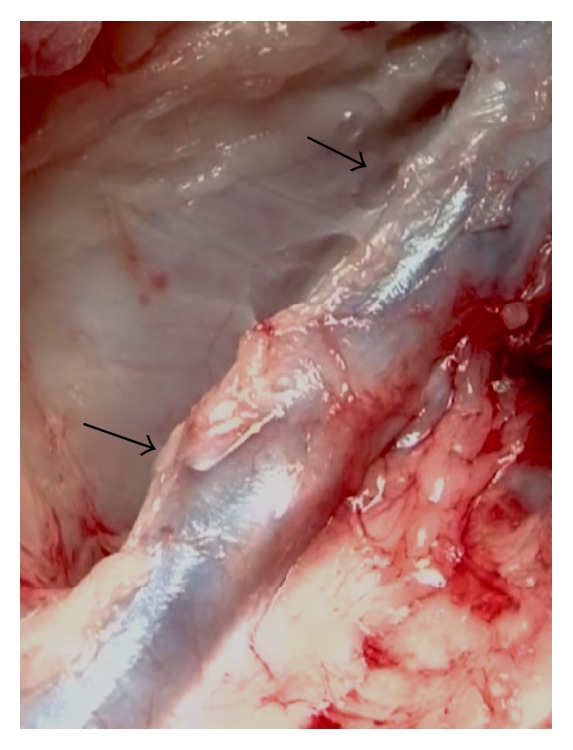
Intraoperative view showing the Petalo CVS implanted in left IJV (arrows). Note the absence of inflammatory reaction of the adventitia and tissue surrounding the vein.

During the standard cutdown of the neck, the isolation of the entire target IJV allowed us to demonstrate the absence of adventitial inflammatory response. The target veins were easily detached from the surrounding tissues due to the absence of fibrosis and adhesions (Figure 5).

**Figure 6:**
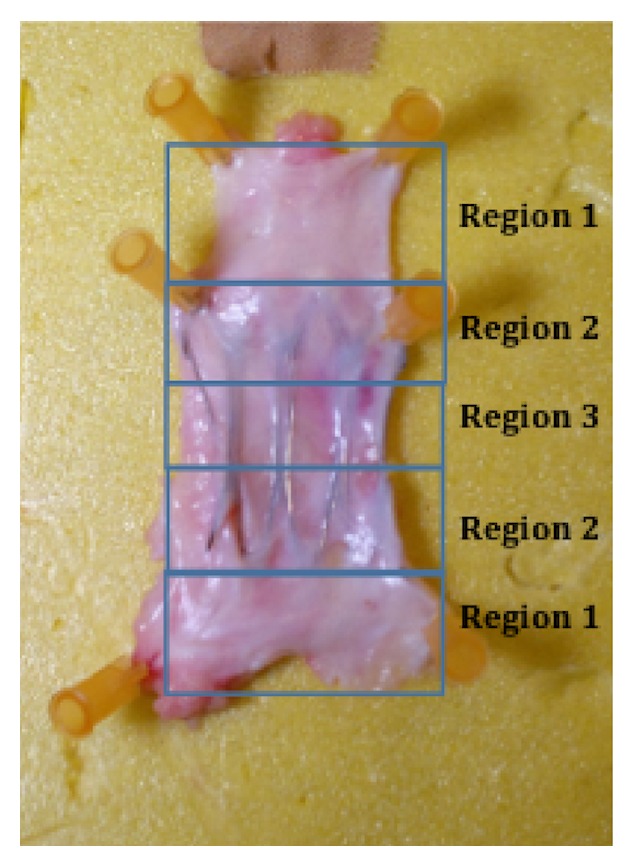
Six-month follow-up. Macroscopic view of the internal surface of a small jugular vein containing Petalo CVS. The device was incorporated in the vein wall mainly at the extremities (Region 2) by a light tissue. Neointimal hyperplasia was absent. Moreover, no fracture of the support modules or transversal bridges of the scaffold were documented. Presence of thrombus was not noted. Region 1: control area; region 2: distal device area; and region 3: middle device area.

No fractures or perforations of the target vein were documented. The target IJVs, including the scaffolds, maintained the physiological compliance. In all target IJVs, the variation of vein diameter by the means of partial collapse of the vein wall into the free modules of the device in response to flow volume and pressure changes and body position were documented (Video 3). After the removal of the target IJVs including the device, the vessel was opened longitudinally and then evaluated (Figure 6).

**Figure 7:**
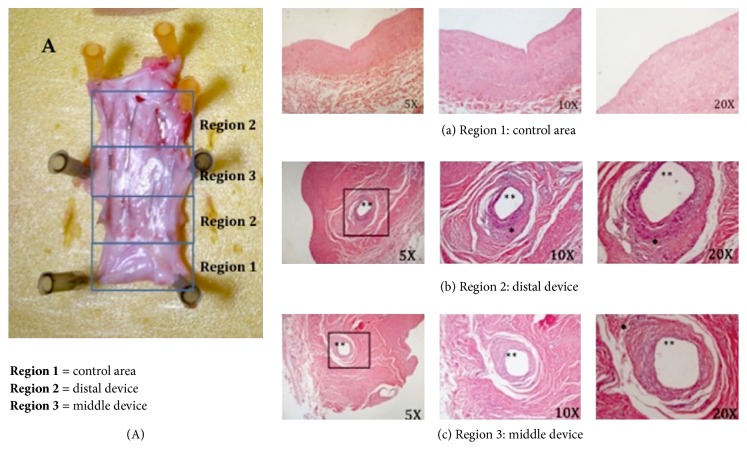
Morphological analyses of internal jugular veins implanted with Petalo CVS for 6 months. Six months after the implant, the swine were sacrificed and IJV were collected for morphological analyses. In (A), gross luminal view of the stent-grafted IJV at 6 months is shown. Representative histological images (hematoxylin-eosin stain) of control (a), distal (b), and middle (c) regions of the IJV are shown. ^*∗∗*^Empty spaces before being occupied by metallic rods. ^*∗*^Inflammatory area.

The devices were incorporated with a light tissue in the vein wall mainly at the extremities, without signs of endoluminal thrombus. No fracture of the support modules or the transversal bridges of the scaffold was documented. The gross anatomy of the vein wall distally and proximally to the device was normal. The portion of the vein between the modules showed an intact endothelial layer and absence of fibrosis. Histological examination revealed only minimal lesions characterized by villous proliferation of the endothelial surface and subendothelial fibrosis, with minimal or absent inflammatory reaction surrounding the inglobated metallic rods. Inflammatory response was indicated by the presence of fibrocytes and, in some cases, by rare inflammatory cells typical of chronic processes (lymphocytes and macrophages). Finally, the thickness of the wall and the tissue covering the device in the distal region was comparable to that of the sections obtained at 3 and 6 months after the procedure (Figure 7).

## 5. Discussion

Obstructive venous diseases, such as hypoplasia, intraluminal defects, and extrinsic compression, may have different hemodynamic effects on the venous system depending on the location, extent, severity, and lack of compensation through collateral pathways [6–10]. Endovenous angioplasty with stenting of iliac vein shows a good safety profile of >90% in nonthrombotic limbs and >80% in thrombotic limbs [11–16]. Venous sinus stenting to reduce intracranial pressure in idiopathic intracranial hypertension results in a significant improvement of neurological outcomes, including headache and cognitive dysfunction [17, 18].

Stenosis due to endoluminal anomalies in the form of malfunctioning valves, septa, or membranes was documented in the IJV tract close to the junction with the subclavian vein and the outlet into the brachiocephalic vein [6]. Recently, IJV stenosis has been treated using endovascular treatment (either PTA or stenting), with positive results in terms of neurological outcomes and/or effects on the quality of life [19–24]. Nevertheless, angioplasty of IJVs is complicated by a high rate of early stenotic recurrence, with incidence of restenosis between 30% and 50% of cases [23–25]. Such high restenosis rates suggest that discussion of inadequate treatment may be more appropriate rather than restenosis or recurrence. Otherwise, stenting of IJVs showed a good efficacy profile with no immediate increase of risk. However, long-term results are not encouraging due to high rate of restenosis [24, 25]. The current design of stents for the venous system shows improved and continuous radial force and resistance to compression. The increasing diameter of vessel toward the heart requires an oversized stent diameter to avoid migration. Permanent overdilatation determines the peripheral slowdown of the flow and the loss of the physiological compliance of the vein [6]. This new device was designed with full consideration of the “collapsible” nature of the vein, which is responsible for great variation in venous capacity with little change in venous pressure. A major change in volume with minimal pressure change is largely due to the cross section of the venous lumen; the elliptical contour at low transmural pressures becomes circular at high transmural pressures [6]. The elliptical cross section in a partially collapsed state has far more resistance than the circular cross section; the vein is distended and resistance falls, allowing an increase in blood flow with little increase in the energy gradient. Little increase in pressure is required to convert a low-volume elliptical tube into a high-volume circular tube (e.g., increase of transmural pressure from 0 to 15 mmHg increases the volume of vein for more than 250%). However, more pressure is required to stretch the venous wall once the circular configuration has been reached [6]. Hence, mechanically paradoxical venous system with unique condition of “venous pressure and venous volume may decrease as flow increases and may increase as flow decreases or reverses” (cf. arterial system; pressure, volume, and flow change in the same direction) can fulfill its role as energy-efficient storage facility of blood [6].

Petalo CVS was developed considering the main characteristics of the vein, that is, the delicate and elastic venous wall and the capacity to adapt its cross-sectional area to flow volume and pressure changes.

Petalo CVS promotes only minimal response of the vein wall, with a primary patency of 100% in scheduled phase 2 procedures. The patency of the target veins was maintained without the need of any specific anticoagulant therapy. The shape and configuration of this device avoid migration outside the target veins and fractures of the scaffold, despite the light structure and reduced radial force. The reported minor proximal migration rate of 20% confined to IJV segment was probably a consequence of larger diameters of the vein wall at the IJV bulb.

The restriction policy of the Italian Minister of Health to prevent animal suffering did not allow us to perform a comparative study with conventional stents. However, the aim of this preclinical study was to evaluate the safety and efficacy of this new venous scaffold in a healthy animal model, without presumption of superiority to conventional stents.

Petalo CVS was implanted at the valve site of healthy IJVs; thus, the efficacy of this device in cases of chronically occluded and scarred veins was not documented. However, the documented precise and stable positioning, control of valve leaflet movement, and absence of fracture of the scaffold, and, finally, the minimal inflammatory response of the vein wall could encourage the use of Petalo CVS in the treatment of venous obstructive diseases.

## 6. Conclusions

This study documented a favourable safety profile of Petalo CVS, a novel endovenous device based on specific concepts. The novel configuration of the scaffold and the respect of the compliance of venous wall provided an excellent patency, even without the use of anticoagulant drugs.

## Figures and Tables

**Table 1:** Baseline characteristics and operative details of the study population, comparing the two different devices used for the study.

	Stent 1 (14–12 × 38 mm)	Stent 2 (14–12 × 48 mm)	Significance (*P* value)
Devices, *n* (%)	21 (65.6)	11 (34.4)	NA
Right IJV devices, *n* (%)	10 (31.2)	6 (18.8)	0.886^*∗*^
Left IJV devices, *n* (%)	11 (34.49)	4 (12.5)	0.886^*∗*^
Anterior IJV devices, *n* (%)	0 (0.0)	1 (3.2)	NA
Middle IJV device implantation	14 (43.8)	8 (25.0)	0.991^*∗*^
Proximal IJV device implantation	7 (21.9)	3 (9.3)	0.991^*∗*^
Device implantation > 1			
(i) RIJV	2	1 (in overlapping with stent 1)	NA
(ii) LIJV	4		
Death, *n* (%)	0 (0.0)	0 (0.0)	NA
Reinterventions, *n* (%)	0 (0.0)	0 (0.0)	NA
Stent graft fracture, *n* (%)	0 (0.0)	0 (0.0)	NA
Inner IJV stent graft migration, *n* (%)	3 (9.3)	3 (9.3)	0.714^*∗*^
Major IJV stent graft migration, *n* (%)	0 (0.0)	0 (0.0)	NA
1-month patency, *n* (%)	21/21 (100)	11/11 (100)	1.000^*∗*^
3-month patency, *n* (%)	10/10 (100)	7/7 (100)	1.000^*∗*^
6-month patency, *n* (%)	6/6 (100)	5/5 (100)	1.000^*∗*^

IJV: internal jugular vein; RIJV: right internal jugular vein; LIJV: left internal jugular vein; ^*∗*^*P* value determined using a Chi-square test. Pearson's Chi-squared test with Yates' continuity correction.

## Supplementary Materials

Supplementary Materials Video 1: procedure of device delivery into the left internal jugular vein. After accessing the venous system through a percutaneous antegrade approach of the right common femoral vein, the left internal jugular vein was cannulated. The selective venography of the internal jugular vein showed a patent vein with a well visible valve apparatus (marked with *∨* on the screen). The device was implanted and released at the site of the valve apparatus (marked with black points) via a standard pull-back stent delivery system. The control phlebography demonstrated the patency of the vein, while the valve apparatus was not clearly visible. The postprocedural echo-color Doppler demonstrated the patency of the vein and the correct positioning of the device. Video 2: six-month follow-up. The selective venography of the right internal jugular vein demonstrated the correct positioning of the device with no signs of fracture and the patency of the vein with a normal contrast medium clearance. The device was perfectly compliant with the vein wall; this is the proof of the concept of the device, which allows the maintenance of the collapsibility and the elasticity properties of the vein wall. Video 3: 6-month follow-up. Intraoperative view. The jugular vein has been dissected before retrieval for histological analysis, and the device is clearly visible inside the vein. Notably, the partial collapse of the vein wall into the free modules of the device allows the adapting of its cross-sectional area to volume changes.
